# The Case of the Positive Pregnancy Pretender: A Potential Cause of Erroneous Test Results in Active-Duty Personnel

**DOI:** 10.7759/cureus.59306

**Published:** 2024-04-29

**Authors:** Jason M Corless, Joseph M Glendening, Kathryn Burtson

**Affiliations:** 1 Internal Medicine, Wright-Patterson Medical Center, Wright-Patterson Air Force Base, USA; 2 Internal Medicine, Wright State University, Dayton, USA; 3 Internal Medicine, Lakenhealth Medical Center, Lakenheath Air Force Base, USA; 4 Internal Medicine, Uniformed Services University of the Health Sciences, Bethesda, USA

**Keywords:** active-duty military personnel, false-positive, weight-loss, beta-human chorionic gonadotropin (β-hcg), pregnancy

## Abstract

The detection of pregnancy is common among those who participate in the care of reproductive-age females. This is especially true in the medical care of active-duty personnel in the armed forces. Considering the impact of a positive urine pregnancy test in this population, it is important to recognize the possibility of false-positive results and their causes. In this case, we explore a false-positive urine pregnancy test due to injectable positive beta-human chorionic gonadotropin (beta-hCG) supplementation used for weight loss. This report concludes that the use of exogenous beta-hCG by physicians and other clinicians should be avoided. Additionally, clinicians should be aware of its use in the community and its possible effect on laboratory testing used to evaluate for pregnancy.

## Introduction

In the armed services’ active-duty population, multiple health factors affect operational readiness and influence the ability to deploy [1]. Pregnancy, often initially evidenced by a positive urine screen, has implications on readiness such as deployability, theater entry, and job-specific training [2].

The confirmation of pregnancy is a common clinical scenario among reproductive-age females within the military and civilian spheres. The diagnosis of pregnancy is typically based on the detection of beta-human chorionic gonadotropin (beta-hCG) either in the urine or blood. Physiologically, beta-hCG is secreted by placental syncytiotrophoblasts and is thought to help maintain the corpus luteum for progesterone production and aid in implantation; beta-hCG levels rise exponentially early in pregnancy along a predictable course and then will decrease gradually during the second and third trimesters [3]. This has led to various available calculations using the difference in beta-hCG levels between multiple measurements as an indicator of a viable pregnancy. For example, most viable intra-uterine pregnancies would be associated with a minimum rise of beta-hCG of 24% in one day and 53% at two days [4,5].

Serum measurements of beta-hCG are a sensitive screening test for pregnancy and allow for the quantification of beta-hCG levels. Most assays determine a positive test result for beta-hCG levels greater than 5 mIU/mL [6]. While detection of serum beta-hCG is very sensitive, there are several identified causes of elevations in the absence of viable pregnancy, including miscarriage, ectopic pregnancy, pituitary beta-hCG production, and trophoblastic disease [7]. This case presents a patient with an incidentally observed positive beta-hCG urine screen and detectable elevations of serum beta-hCG in the absence of pregnancy in the setting of injected beta-hCG for weight loss.

## Case presentation

A 27-year-old active-duty female with a medical history of recurrent nephrolithiasis, polycystic ovarian syndrome, and venous sinus thrombosis presented to the emergency department for right flank pain, dysuria, nausea, and vomiting for three days. The right flank pain was sharp, colicky, and radiated to her groin. On physical exam, her vital signs were within normal limits. Her BMI was 27 kg/m2. The patient had mild right upper quadrant abdominal tenderness without rebound or guarding, and no lower abdominal tenderness. Basic electrolytes and a complete blood count with differential were normal. Urinalysis revealed a large amount of blood and calcium oxalate crystals. The patient was of reproductive age and was screened for pregnancy as part of the initial evaluation. Urine beta-hCG was detected after which serum beta-hCG was obtained and was measured at 7.89 mIU/mL (Table 1).

**Table 1:** Laboratory data Beta-hCG, beta-human chorionic gonadotropin; Na, sodium; K, potassium; Cl, chloride; CO2, carbon dioxide; BUN, blood urea nitrogen; Cr, serum creatinine; WBC, white blood cell count; Hgb, hemoglobin, Urine WBC, urine white blood cells.

Test	Observed value at first encounter	Observed value at second encounter	Reference range
Urine beta-hCG	POSITIVE	POSITIVE	>20mIU/mL
Serum beta-hCG	7.89 mIU/mL	9.20 mIU/mL	0.46-5.42 mIU/mL
Na	137 mmol/L	137 mmol/L	137-145 mmol/L
K	3.8 mmol/L	3.8 mmol/L	3.5-5.1 mmol/L
Cl	107 mmol/L	105 mmol/L	98-107 mmol/L
CO2	19 mmol/L	24 mmol/L	22-30 mmol/L
BUN	5 mg/dL	9 mg/dL	7-17 mg/dL
Cr	0.60 mg/dL	0.80 mg/dL	.52-1.04 mg/dL
Glucose	87 mg/dL	76 mg/dL	74-106 mg/dL
WBC	5.8 x10^3/mcL	8.1 x10^3/mcL	4.6-9.4 x10^3/mcL
Hgb	13.9 g/dL	14.2 g/dL	12.0-16.0 g/dL
Platelets	255 x10(3)/mcL	233 x10(3)/mcL	140-420 x10(3)/mcL
Urine Blood	LARGE	NONE	NONE
Urine WBC	No data	0-5	<11-20
Urine Leukocyte Esterase	NEGATIVE	NEGATIVE	NEGATIVE
Urine Nitrite	POSITIVE	NEGATIVE	NEGATIVE
Urine crystals	Calcium oxalate crystals	Calcium oxalate crystals	None

Further history was obtained regarding the patient’s elevated beta-hCG levels. The patient reported that she was receiving beta-hCG injections as part of a weight loss program prescribed through a local clinic. She did not take any oral contraceptives due to her history of thrombosis nor did she use any form of long-acting reversible contraception such as an intrauterine device. However, she stated there was no possibility that she could be pregnant as she had no history of sexual activity in the last several months. Her last menstrual period was three weeks ago and was unremarkable. She denied any other early signs of pregnancy such as nausea, vomiting, breast enlargement or tenderness, or increased urinary frequency. It was determined that the cause of her elevated serum beta-hCG levels were the exogenous beta-hCG injections.

CT imaging of her abdomen was obtained as the clinical probability of pregnancy was felt to be low with an alternative explanation for her mildly elevated beta-hCG levels. This imaging revealed a three-millimeter stone in the distal right ureter with mild right-sided hydronephrosis. She was diagnosed with nephrolithiasis and discharged from the emergency department with medications for pain and nausea with instructions to monitor for stone passage.

**Figure 1:**
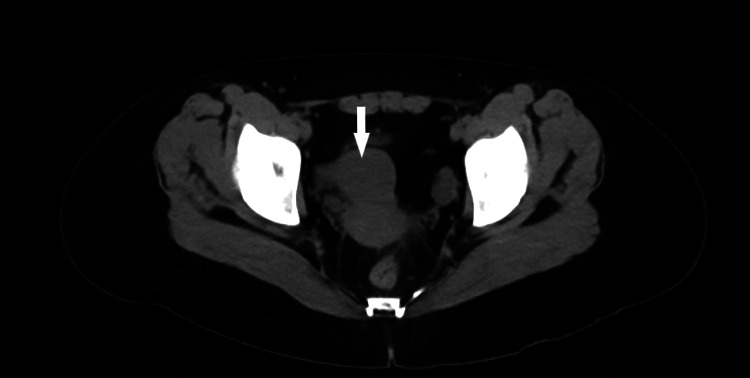
Computed tomography imaging showing lack of a gravid uterus. Arrow showing uterus.

The patient returned to the emergency department after two days due to persistent pain and anorexia. Physical exam was similar to the prior presentation with only mild tenderness to palpation of the right upper abdomen and flank. Laboratory work-up was again notable for a detectable quantitative beta-hCG level. The second level had been measured greater than 48 hours after initial collection and was found to be 9.20 mIU/mL (Table 1). This represented an increase of 1.31 mIU/mL or 16.6%. This increase, in addition to other factors from the patient history noted above, was inconsistent with early pregnancy in which the beta-hCG level would be expected to at least double over this period of greater than 48 hours [4,5]. Repeat CT again revealed nephrolithiasis with mild to moderate hydronephrosis now with mild right perinephric stranding. CT imaging was also significant for a non-gravid uterus further negating the possibility of advanced pregnancy (Figure 1).

The patient was admitted to the medical service with plans for urologic intervention the following morning. She received intravenous fluids, pain medications, and an alpha-antagonist. The patient underwent a successful ureteroscopy and stone retrieval the following morning. She was discharged later that afternoon with outpatient follow-up.

## Discussion

The use of beta-hCG for weight loss began in the 1950s and is typically used in conjunction with severe calorie restricted diets limiting intake to 500 Calories daily. Several physiologic mechanisms were previously suggested for how beta-hCG may lead to weight loss. Physiologic levels of beta-hCG may lead to a period of transient hyperthyroidism observed physiologically during pregnancy. High levels of beta-hCG have also been previously used in the treatment of male hypogonadotropic hypogonadism. The hormone beta-hCG is thought to stimulate the luteinizing hormone (LH) receptor leading to an increase in testosterone production and indirectly affecting fat distribution [8]. However, since that time, current data do not indicate that beta-hCG has any utility as therapy for weight loss [9-12]. The Food and Drug Association (FDA) does not approve of the use of beta-hCG for weight loss and advises consumers using the medication for this purpose to stop using the medication and to discard it promptly [13]. Physicians should not prescribe beta-hCG off-label for the purpose of weight loss and should discourage its use for this purpose. Additional concerns are present concerning other dangers of very low-calorie diets that often accompany beta-hCG use and these diets should also be discouraged.

Despite the paucity of evidence supporting beta-hCG therapy for weight loss, it continues to be used by many patients. This case highlights the importance of clinicians’ awareness of beta-hCG use for weight loss, especially in the military. Prior research has demonstrated that high doses of exogenous beta-hCG, traditionally 5,000 units, intended to induce ovulation can interfere with high-sensitivity pregnancy tests [14]. This case demonstrates that lower doses of exogenous beta-hCG intended for weight loss can lead to a similar result of a positive pregnancy test in the absence of pregnancy. In our case, the absence of pregnancy was determined given the absence of sexual activity and negative imaging. Additionally, the minimal elevations of beta-hCG observed more than 48 hours apart would not be consistent with pregnancy.

Should physicians encounter this clinical situation, a thorough medication and sexual history should be included in the complete evaluation of the patient to determine the likelihood of pregnancy. In the military, this in-depth evaluation should be performed before the active-duty military member is referred to force management and public health personnel to avoid possible duty and deployment restrictions. 

A final consideration relevant to providers in the United States military is that beta-hCG is considered a prohibited substance for service members as outlined in the Department of Defense’s (DoD) resource Operation Supplement Safety (OPSS) [15]. The document, DoD Instruction 6130.06, which establishes policy regarding the use of dietary supplements in the DoD, states that service members can be prosecuted for use of supplements with prohibited ingredients from the OPSS resource [16]. Physicians who care for active-duty patients should be aware of such prohibited substances and counsel patients regarding their use.

## Conclusions

Here we describe an example of false-positive pregnancy testing due to exogenous beta-hCG use for weight loss in an active-duty Airman. This case highlights the importance of a thorough clinical history and physical examination to rule out pregnancy as well as awareness of possible causes of misleading laboratory results. This evaluation is important especially when a patient’s pregnancy status may affect further necessary diagnostic and management decisions as in this patient who required CT imaging for diagnosis of her nephrolithiasis. Early evaluation for pregnancy and possible causes of false-positive results is especially true in the military where pregnancy can cause limitations on duty and deployment and where there are restrictions on the use of certain substances and supplements. Finally, beta-hCG use has not been found to be effective for weight loss. It is the responsibility of physicians to be aware of beta-hCG use as a possible cause of a false-positive pregnancy screen as well as to discourage its use for weight loss.
